# The *C. elegans* VAPB homolog VPR-1 is a permissive signal for gonad development

**DOI:** 10.1242/dev.152207

**Published:** 2017-06-15

**Authors:** Pauline A. Cottee, Tim Cole, Jessica Schultz, Hieu D. Hoang, Jack Vibbert, Sung Min Han, Michael A. Miller

**Affiliations:** Department of Cell, Developmental and Integrative Biology, University of Alabama at Birmingham, Birmingham, AL 35294, USA

**Keywords:** MSP, ALS, Gonad, *Caenorhabditis elegans* development, VAPB, Signaling

## Abstract

VAMP/synaptobrevin-associated proteins (VAPs) contain an N-terminal major sperm protein domain (MSPd) that is associated with amyotrophic lateral sclerosis. VAPs have an intracellular housekeeping function, as well as an extracellular signaling function mediated by the secreted MSPd. Here we show that the *C. elegans* VAP homolog VPR-1 is essential for gonad development. *vpr-1* null mutants are maternal effect sterile due to arrested gonadogenesis following embryo hatching. Somatic gonadal precursor cells and germ cells fail to proliferate fully and complete their respective differentiation programs. Maternal or zygotic *vpr-1* expression is sufficient to induce gonadogenesis and fertility. Genetic mosaic and cell type-specific expression studies indicate that *vpr-1* activity is important in the nervous system, germ line and intestine. VPR-1 acts in parallel to Notch signaling, a key regulator of germline stem cell proliferation and differentiation. Neuronal *vpr-1* expression is sufficient for gonadogenesis induction during a limited time period shortly after hatching. These results support the model that the secreted VPR-1 MSPd acts at least in part on gonadal sheath cell precursors in L1 to early L2 stage hermaphrodites to permit gonadogenesis.

## Abstract (precis)

**Highlighted Article:** *vpr-1* null mutants are sterile upon hatching, a defect rescued by the expression of MSPd from almost any tissue except for the somatic gonad itself. See also the companion paper by Schultz et al.

## INTRODUCTION

The major sperm protein domain (MSPd) is an evolutionarily conserved immunoglobulin-like structure found in unicellular and multicellular eukaryotes (Lev et al., 2008; Miller et al., 2001; Tarr and Scott, 2005). The name derives from nematode sperm, which synthesize copious amounts of major sperm proteins (MSPs) during spermatogenesis (Klass and Hirsh, 1981). Sperm MSPs function as cytoskeletal elements and secreted signaling molecules (Ellis and Stanfield, 2014; Han et al., 2010). During spermiogenesis, cytosolic MSPs form extensive filament networks in the pseudopod that drive translocation (Roberts and Stewart, 2012; Smith, 2006). MSPs are also secreted into the extracellular environment by a vesicle budding mechanism (Kosinski et al., 2005; Miller et al., 2001). Secreted MSPs bind to the VAB-1 Eph receptor and other receptors expressed on oocyte and gonadal sheath cell membranes (Brisbin et al., 2009; Cheng et al., 2008; Miller et al., 2003); this binding modulates G-protein signaling in sheath cells that triggers oocyte meiotic maturation and sheath contraction (Govindan et al., 2006).

**Fig. 1.**
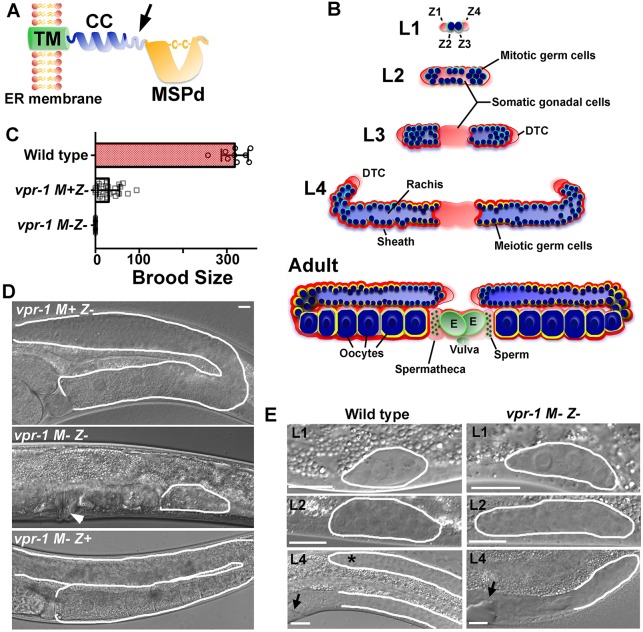
***C. elegans vpr-1* null mutants are maternal effect sterile.** (A) VAP structure showing major sperm protein domain (MSPd), coiled-coil motif (CC) and transmembrane domain (TM). VAPs are type II membrane proteins with the TM spanning the endoplasmic reticulum (ER) and MSPd in the cytosol. Arrow indicates approximate (unknown) site of proteolytic processing, which liberates the MSPd for secretion. (B) Postembryonic gonad development showing larval stages (L1-L4) and adult structure. The gonad primordium in freshly hatched embryos consists of the germline precursors Z2 and Z3 (dark blue) and the somatic gonad precursors Z1 and Z4 (pale red). The distal tip cell (DTC) migrates during larval development to form the U-shaped gonad arms. The DTC also expresses the Notch ligand LAG-2, which acts through the GLP-1 receptor to control germ cell proliferation and meiotic entry (Hansen and Schedl, 2013). Germ cells enter meiosis (blue circles with yellow outline) during the L4 stage, forming sperm first and then oocytes in adulthood. Sperm are stored in the spermatheca and embryos (green, E) in the uterus. (C) Average brood sizes of wild-type and *vpr-1* mutant hermaphrodites lacking zygotic (*Z−*) or maternal and zygotic (*M− Z−*) expression. Error bars are s.d. *N*=9 for wild type, *N*=26 for *vpr-1 M+ Z−* and *N*=50 for *vpr-1 M− Z−*. (D) DIC images of *vpr-1* mutant adult hermaphrodite gonads. Arrowhead points to the vulva, which is induced by the anchor cell. (E) DIC micrographs of wild-type and *vpr-1* mutant gonads during larval stages. Asterisk indicates the elongating distal gonad arm. Arrows point to developing vulva (left). Gonads are outlined in white. Scale bars: 10 µm.

Sperm MSPs contain a single MSPd that is related to the N-terminal MSPd from VAMP/synaptobrevin-associated proteins (VAPs) (Fig. 1A) (Lev et al., 2008; Skehel et al., 1995). Although sperm MSPs appear to be unique to nematodes, VAPs are widely conserved among animal species. Mammals have two VAP paralogs called VAPA and VAPB, which both contain an N-terminal MSPd. Mutations in the VAPB MSPd are associated with amyotrophic lateral sclerosis (ALS) and spinal muscular atrophy (SMA), two motor neuron degeneration diseases (Nishimura et al., 2004). VAPs are broadly expressed type II transmembrane proteins that function as scaffolding components at intracellular membrane contact sites, such as those bridging the endoplasmic reticulum and mitochondria or peroxisomes (Costello et al., 2017; Dong et al., 2016; Gomez-Suaga et al., 2017; Hua et al., 2017; Lev et al., 2008; Stefan et al., 2011; Stoica et al., 2016). This cell-autonomous activity affects actin nucleation, endocytic trafficking, lipid transfer, Ca^2+^ dynamics and other processes. In addition, the VAP MSPd is cleaved from the transmembrane domain and secreted in a cell type-specific fashion (Deidda et al., 2014; Han et al., 2013, 2012; Tsuda et al., 2008). The secreted MSPd binds to Eph protein tyrosine kinase and Lar-like protein tyrosine phosphatase receptors, which are important for neuron development and striated muscle energy metabolism, respectively (Han et al., 2013, 2012; Tsuda et al., 2008). Hence, VAPs also have an important non-cell-autonomous signaling function.

To better understand essential roles of animal VAPs, we have been studying the nematode model *C. elegans.* The *C. elegans* genome encodes numerous proteins that contain an MSPd (Tarr and Scott, 2005). Only *vpr-1* encodes the N-terminal MSPd, coiled-coil motif and single transmembrane region characteristic of VAPs (Fig. 1A) (Miller et al., 2001; Tsuda et al., 2008). *C. elegans vpr-1* mutants share phenotypes in common with *Drosophila Vap* (*Vap33*) and mouse *Vapb* mutants (Han et al., 2013, 2012; Larroquette et al., 2015; Tsuda et al., 2008). Moreover, these VAPs are functionally interchangeable and their MSPds have conserved signaling and receptor binding activities (Han et al., 2012; Lua et al., 2011; Tsuda et al., 2008). *vpr-1* null mutants exhibit incompletely penetrant embryonic lethality and 100% sterility, whereas *Drosophila Vap* null mutants die as second or third instar larvae (Pennetta et al., 2002; Tsuda et al., 2008). By contrast, mouse *Vapb* mutants are viable and fertile (Kabashi et al., 2013; Larroquette et al., 2015), perhaps due to redundancy or division of function with *Vapa.*

Here we characterize the role of *vpr-1* in gonadogenesis. *vpr-1* null mutants are maternal effect sterile due to arrested somatic gonadal precursor cell and germ cell development. We show that *vpr-1* expression is crucial in neurons and germ cells to induce gonadogenesis. Moreover, transgenic *vpr-1* overexpression is sufficient for gonadogenesis induction in several cell types accessible to the pseudocoelom, a primitive circulatory cavity. The most likely target tissue is the sheath cell precursors, which are required for germline proliferation and differentiation independent of GLP-1 Notch receptor signaling (Killian and Hubbard, 2005; McCarter et al., 1997; Sulston et al., 1983). Consistent with this idea, *vpr-1* acts independently of *glp-1* at a stage when sheath precursors are starting to divide. Collectively, our data support the model that the VPR-1 MSPd acts as a permissive signal for gonadogenesis early in postembryonic development.

## RESULTS

### *vpr-1* null mutants are maternal effect sterile

The *C. elegans* hermaphrodite gonad develops postembryonically from a primordium consisting of two somatic gonadal precursor cells, Z1 and Z4, and two germline precursors, Z2 and Z3 (Fig. 1B) (Hubbard et al., 2013; Pazdernik and Schedl, 2013). During larval development, Z1 and Z4 descendants form the distal tip cells (DTCs), anchor cell, gonadal sheath cells, spermathecae and uterus. Z2 and Z3 start proliferating during L1, forming a germline syncytium with a central core, called the rachis, during subsequent larval stages (Fig. 1B) (Amini et al., 2014). A stem cell population at the distal gonad tip gives rise to sperm and oocyte precursors (Kimble and Crittenden, 2005). Sperm develop first at the L4 stage, followed by oocytes during early adulthood (L'Hernault, 2006; Marcello et al., 2013). Germ cell development and meiosis proceed from the distal to proximal ends (Fig. 1B). As a result, the adult hermaphrodite has two U-shaped gonad arms that connect to a common uterus.

The *vpr-1(tm1411)* null mutation eliminates the first two *vpr-1* exons, which encode the MSPd and part of the coiled-coil motif (Tsuda et al., 2008). Homozygous F1 *vpr-1(tm1411)* mutants derived from P0 *vpr-1(tm1411)/hT2* heterozygous hermaphrodites exhibit limited fertility, with an average brood size of ∼30 F2 progeny (Fig. 1C). These progeny give rise to completely sterile *vpr-1(tm1411)* hermaphrodites lacking maternal (*M−*) and zygotic (*Z−*) *vpr-1* expression (Fig. 1C). F1 *vpr-1(tm1411)* mutant adults with maternal *vpr-1* mRNA (*M*+ *Z−*) contain functional sperm and oocytes (Fig. 1D). By contrast, *M− Z− vpr-1* mutant adults contain stunted gonads without mature gametes (Fig. 1D). To investigate gonad development, we monitored staged animals using differential interference contrast (DIC) microscopy. The 4-cell gonad primordium appears similar in freshly hatched wild-type and *vpr-1* mutant hermaphrodites (Fig. 1E). However, the distal portion of each gonad stops developing in *vpr-1* mutants (Fig. 1E). We mated wild-type males to *M+ Z− vpr-1* mutant hermaphrodites to test whether zygotic *vpr-1* expression is sufficient to induce gonadogenesis. Male sperm provide a wild-type *vpr-1* copy to oocytes completely lacking *vpr-1*. The *M− Z+ vpr-1(tm1411)/+* progeny produce functional gametes, similar to wild-type and *M+ Z− vpr-1* mutant hermaphrodites (Fig. 1D). We conclude that *vpr-1* is a maternal effect sterile gene and that maternal or zygotic *vpr-1* expression is sufficient for gonadogenesis.

### *vpr-1* loss arrests germ cell and somatic gonadal precursor cell development

**Fig. 2.**
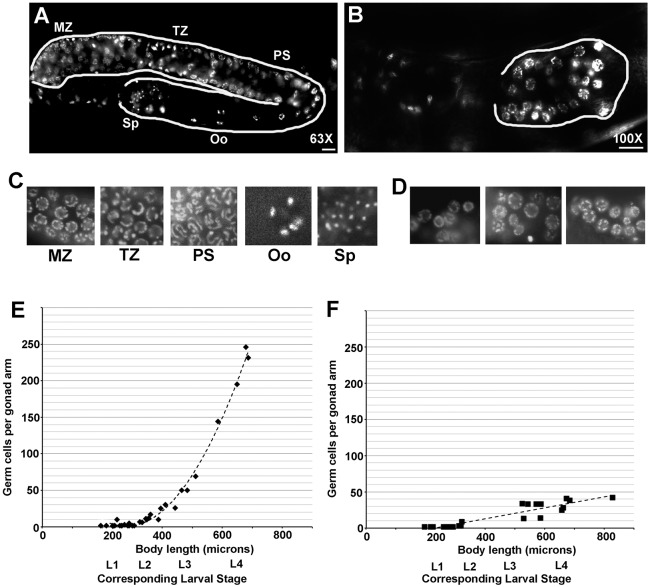
***vpr-1* is essential for** **germ cell expansion during larval development.** (A,B) Adult wild-type (A) and *vpr-1 M− Z−* (B) hermaphrodites stained with the DNA dye DAPI to identify germ cell stages. Gonads are outlined in white. In wild-type gonads, mitotic zone (MZ) precursor nuclei are observed at the distal end. From the distal to proximal end, meiotic transition zone (TZ) nuclei, pachytene stage (PS) nuclei, and spread diakinesis stage chromosomes of oocyte (Oo) nuclei are indicated. Highly condensed sperm (Sp) chromatin is seen at the most proximal end. Differentiating germ cells are not observed in *vpr-1 M− Z−* gonads (B). Scale bars: 10 µm. (C,D) DAPI staining of dissected wild-type (C) and *vpr-1 M− Z−* (D) gonads improves imaging of germ cell chromosome morphology. Three different *vpr-1 M− Z−* gonads are shown. (E,F) Counting germ cell numbers during wild-type larval development (E) shows exponential expansion that is not observed in *vpr-1 M− Z−* larva (F). Germ cell number was counted in DAPI-stained larva from 1 μm *z*-stacks imaged through the body. Total germ cell number was divided by two in cases where both anterior and posterior gonads were counted (nearly all cases). Differentiating spermatocytes were not included in L4 stage wild-type gonads and are not observed in *vpr-1* null gonads. Larval development is represented on the *x*-axis as total worm body length instead of time post-hatching because *vpr-1 M− Z−* hermaphrodites grow more slowly than wild-type hermaphrodites (Han et al., 2013, 2012). Dotted line indicates best-fit polynomial equation.

*M− Z− vpr-1* mutant adult gonads are much smaller than control adult gonads, suggesting that germ cell development is abnormal. To investigate germ cells, we used DAPI to assess chromosome morphology. In the wild type, germ cell chromosomes exhibit characteristic features in the mitotic, meiotic transition, and meiotic pachytene zones (Fig. 2). Mature sperm have highly condensed chromosomes, whereas growing oocytes in diakinesis have dispersed chromosomes (Fig. 2A,C). *M− Z− vpr-1* mutant adult gonads contain fewer than 80 germ cells and are without sperm or oocytes (Fig. 2B,D). Germ cell chromosomes more closely resemble those in the mitotic or transition zone, although chromosome morphology is often abnormal (Fig. 2D). Next, we counted the number of germ cell nuclei during larval stages. Wild-type gonads exhibit a robust increase in germ cell numbers at L3 and L4 stages (Fig. 2E), which is due to GLP-1 Notch receptor signaling and a parallel pathway requiring gonadal sheath cell precursors (Killian and Hubbard, 2005; Kimble and Crittenden, 2005; McCarter et al., 1997). In contrast to the wild type, *vpr-1* null mutant gonads fail to expand the germ line during larval development (Fig. 2F). These data indicate that *vpr-1* is essential for germ cell expansion and differentiation.

**Fig. 3.**
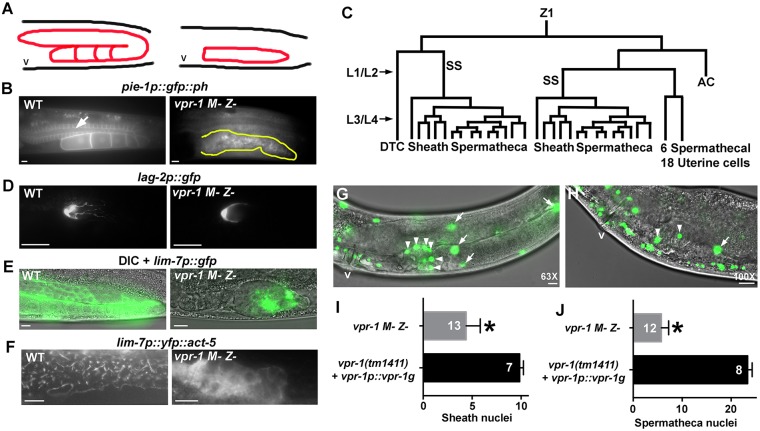
***vpr-1* is essential for postembryonic somatic gonad development.** (A) Diagrams of gonad orientation and structure in wild-type (left) and *vpr-1 M− Z−* (right) hermaphrodites. (B) The germline plasma membrane GFP::PH marker (Audhya et al., 2005) reveals overall gonad structure in transgenic wild type (WT) and *vpr-1* mutants. WT distal gonads contain a central cytoplasmic core called the rachis (arrow), which shares germ cell cytoplasmic contents. A *vpr-1* mutant gonad (outlined in yellow) shows disorganized structure and poorly defined or absent rachis. (C) Diagram showing somatic gonad lineages (Z1 is shown) that form the DTC, 10 sheath cells, 24 spermathecal cells, 18 uterine cells, and the anchor cell (AC). The AC is necessary for vulva development (Hubbard and Greenstein, 2000). Z4 forms the other gonad. Approximate L1/L2 and L3/L4 transition periods are indicated. (D) Transgenic WT and *vpr-1* mutants expressing GFP in the DTC driven by the *lag-2* Notch ligand promoter (Blelloch et al., 1999). (E) Transgenic WT and *vpr-1* mutants expressing GFP in the gonadal sheath cells driven by the *lim-7* promoter (Hall et al., 1999). (F) Transgenic WT and *vpr-1* mutants expressing YFP::ACT-5 actin in the sheath cells (Kinchen et al., 2005). Note that the actin filaments seen in WT distal sheath cells are not observed in *vpr-1* mutant sheath cells. (G-J) The *sur-5p::NLS-GFP* transgenic reporter, which expresses GFP in the nuclei of all somatic cells (Yochem et al., 1998), was used to visualize and quantify somatic gonadal cells from 1 μm *z*-stacks in live young adult animals. (G) A single *z*-stack image showing numerous spermathecal cell (arrowheads) and sheath cell (arrows) nuclei in *vpr-1(tm1411)* null mutants expressing a transgene containing the *vpr-1* genomic locus (fosmid DNA). (H) A similar image from *vpr-1 M− Z−* gonads shows fewer spermatheca and sheath nuclei. Gonad orientation is shown in A. Quantification of sheath nuclei (I) and spermatheca nuclei (J) from the *z*-stacks. White numbers indicate number of gonads scored. Error bars are s.d. *P<0.001 (Student's *t*-test). DTC, distal tip cell; SS, sheath/spermatheca precursors; v, vulva. Scale bars: 10 µm.

To examine gonad architecture, we used the *pie-1p::gfp::ph* transgene, which drives germline expression of a GFP::PH fusion protein that acts as a plasma membrane marker (Audhya et al., 2005). In the wild type, germ cells in the distal gonad surround and open into the rachis, which forms during larval development (Amini et al., 2014). A well-defined rachis is not observed in *M− Z− vpr-1* mutant gonads (Fig. 3A,B). Instead, germ cells with fully enclosed plasma membranes are disordered throughout the core region. We previously showed that *vpr-1* loss causes DTC migration failure (Tsuda et al., 2008). The DTCs are generated from Z1 and Z4 divisions during L1 (Fig. 3C) and start migrating along the ventral basement membrane during L2, before turning 180° in L3 (Hubbard and Greenstein, 2000). We used the *lag-2p::gfp* transgene to monitor DTC development in *vpr-1* mutants (Byrd et al., 2014). In *M− Z− vpr-1* mutant gonads, the DTCs are similar in morphology to those in control L2 or L3 gonads, although they lack the membranous processes seen in adult gonads (Fig. 3D). The vulva, which is induced by the anchor cell during the L3 stage, appears to form normally (Fig. 1D,E). Similar to the DTCs, the anchor cell is derived from Z1 or Z4 divisions during L1 and early L2 (Fig. 3C). Thus, two of the earliest differentiating somatic gonadal cell types, namely the DTCs and anchor cell, appear to form during early larval development in *vpr-1* mutants.

Gonadal sheath cells arise from sheath/spermatheca (SS) blast cells formed during L1 and early L2 (Fig. 3C) (Hubbard and Greenstein, 2000). To investigate sheath cells, we used the *lim-7p::gfp* transgene (Hall et al., 1999). In adult controls, GFP is observed in the four most distal sheath cell pairs, tightly encircling the germ cells and developing oocytes (Fig. 3E). We observe GFP expression in *vpr-1* null gonads, but sheath cells fail to completely cover the germ cells, suggesting that sheath proliferation and development are incomplete (Fig. 3E). Further support comes from the *lim-7p::yfp::act-5* transgene, which labels the sheath cell actin cytoskeleton (Kinchen et al., 2005). *vpr-1* mutant sheath cells do not exhibit the actin filament organization seen in control sheath cells (Fig. 3F). SS blast cells give rise to 10 sheath cells and 18 spermathecal cells from early L3 to mid-L4 stages (Fig. 3C). The adult spermatheca comprises 24 cells in total, which derive from SS and a sister lineage that also forms the uterus (Fig. 3C) (Hubbard and Greenstein, 2000). We used the *sur-5p::NLS-GFP* marker that expresses nuclear GFP in all somatic cells (Yochem et al., 1998) and serial imaging in 1 μm *z*-stacks to count the number of sheath and spermathecal cells in young adult control and *vpr-1 M− Z−* gonads (Fig. 3G-J). *vpr-1(tm1411)* null mutants transgenically expressing *vpr-1* from fosmid DNA contain ∼10 sheath cells (Fig. 3G,I) and 24 spermathecal cells per gonad (Fig. 3G,J). By contrast, *vpr-1 M− Z−* gonads contain severely reduced numbers of sheath (∼4) and spermathecal (∼6) cells (Fig. 3H-J). The uterine lineage is also likely to be affected, although we did not count these cells. We conclude that *vpr-1* is essential for proliferation and differentiation of somatic gonadal precursors that form sheath and spermathecal lineages. These precursors are essential for germ cell expansion and differentiation (Killian and Hubbard, 2005; McCarter et al., 1997).

### Zygotic *vpr-1* is important in the germ line, nervous system and intestine

*vpr-1* is broadly expressed in most tissues (Tsuda et al., 2008). We used genetic mosaic analysis to investigate where *vpr-1* functions (i.e. in which cell type) to promote gonad development. Transgenic *vpr-1(tm1411)* null mutants were generated using a DNA fosmid containing the *vpr-1* genomic locus and a plasmid containing the *sur-5p::NLS-GFP* lineage marker (Yochem et al., 1998). Transgenic DNA is spontaneously lost at low frequency during cell division. When a loss occurs early in development, mosaic worms are generated. Taking advantage of the invariant *C. elegans* embryonic lineage, *vpr-1* mosaic worms were created with the *vpr-1^+^* fosmid eliminated in the germ line, somatic gonadal cells, body wall muscles, intestinal cells or neurons.

**Fig. 4.**
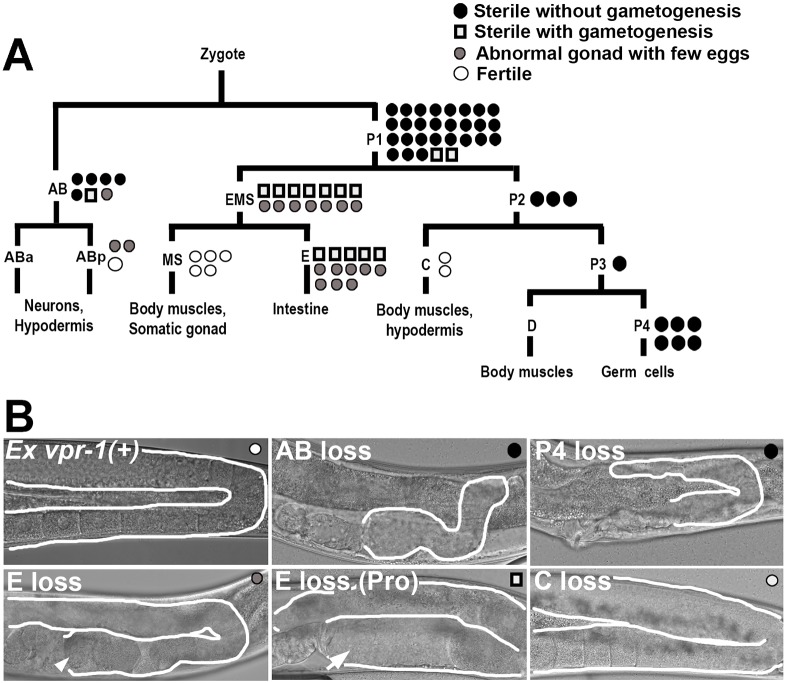
***vpr-1* acts primarily in the germ line and nervous system to promote gonadogenesis.** (A) Genetic mosaic analysis showing cell lineages of major tissues. Each circle or square indicates one genetic mosaic worm. Points at which the genomic copy of *vpr-1(+)* was lost and the resulting phenotype are indicated. (B) Representative DIC images of gonads in *vpr-1(tm1411)* mosaic worms. *Ex vpr-1(+)* indicates expression of the *vpr-1* genomic locus via an extrachromosomal array. Gonads are outlined in white. Arrowhead indicates an endomitotic oocyte that failed to ovulate and the arrow indicates a proximal germ cell tumor (Pro phenotype). Both phenotypes can arise from abnormal somatic gonad development or function (Hubbard and Greenstein, 2000; Killian and Hubbard, 2005).

Expressing the *vpr-1* genomic locus in *vpr-1(tm1411)* worms rescued the gonad defects (Fig. 4) in three independent transgenic lines. These lines were maintained as transgenic *vpr-1* mutant homozygotes. Importantly, complete transgene loss in F1 progeny resulted in sterile adults, indicating that maternal *vpr-1* expression from the extrachromosomal arrays was insufficient for gonadogenesis. Thus, the mosaic lines provide a readout of zygotic *vpr-1* activity only. The loss of *vpr-1* from the AB lineage, which gives rise to the nervous system, or the P lineages, which give rise to the germ line, caused arrested gonadogenesis (Fig. 4). These gonads often appeared larger than *M− Z− vpr-1* gonads (Figs 1–3), but rarely contained visible oocytes. *vpr-1* loss from the MS lineage, which produces the somatic gonad (i.e. Z1, Z4, and their descendants; Fig. 3C), or C lineage, which gives rise to specific body wall muscle and hypodermal cells, did not disrupt development (Fig. 4). *vpr-1* loss from the EMS and E lineages that form the intestine did not prevent germ cell expansion or gamete differentiation. However, 12/27 mutants were sterile due to either ectopic germ cell proliferation in the proximal gonad (i.e. the Pro phenotype) or failed ovulation, two defects consistent with abnormal somatic gonad development or function (Fig. 4) (Hubbard and Greenstein, 2000). The remaining 15/27 mutants produced a small number of fertilized eggs. These mosaic data suggest that zygotic *vpr-1* expression from the germ line, nervous system and intestine is important for fertility. Therefore, multiple tissue sources contribute *vpr-1* genetic activity that promotes gonad development.

### *vpr-1* can induce gonadogenesis non-cell-autonomously

**Fig. 5.**
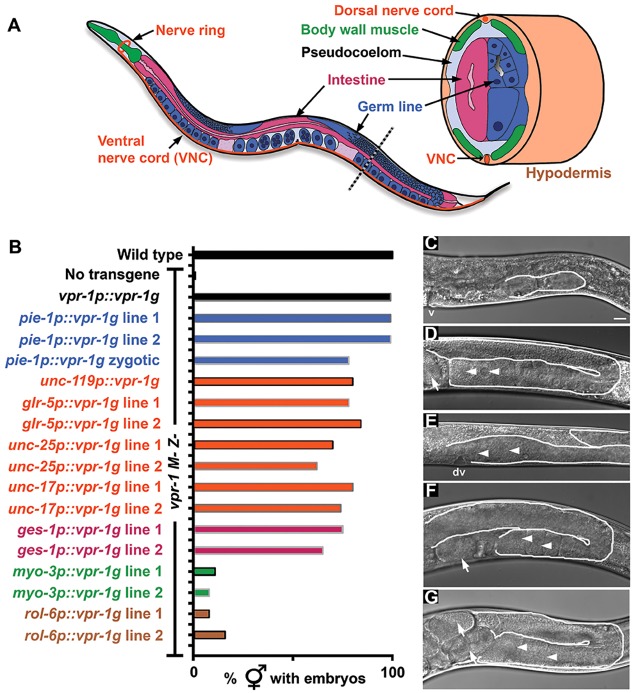
**Transgenic *vpr-1* expression in diverse cell types is sufficient to rescue the *vpr-1 M− Z−* gonadogenesis defect.** (A) Diagram of adult hermaphrodite showing major tissues (adapted from Altun and Hall, 2017). The gonad is not innervated and the nervous system, intestine and gonad are separated by the pseudocoelom, a primitive circulatory cavity. (B) Quantification of adult control and transgenic *vpr-1* mutant hermaphrodites containing fertilized eggs. The *vpr-1* promoter drives expression in most cell types, the *pie-1* promoter drives expression in the germ line, the *unc-119* promoter drives expression throughout the nervous system, the *glr-5* promoter drives expression specifically in ∼56 head interneurons, the *unc-25* promoter drives expression specifically in 26 GABAergic motor neurons, the *unc-17* promoter drives expression specifically in ∼80 cholinergic neurons, the *ges-1* promoter drives expression specifically in the intestine, the *myo-3* promoter drives expression specifically in body wall muscle, and the *rol-6* promoter drives expression specifically in the hypodermis. Lines are color-coded in accordance with A to help illustrate expression patterns. (C-G) DIC images of *vpr-1* mutant control (C) and transgenic *vpr-1* mutant gonads expressing *vpr-1* in the germ line (D), zygotic germ line (E), nervous system (F) and head interneurons (G). Note in E that ∼20% of transgenic worms with zygotic germline *vpr-1* expression show oocyte development next to the developing vulva (dv), instead of the spermatheca. In these cases, the spermatheca and uterus exhibit delayed development relative to the germ line. Arrowheads indicate oocytes and arrows indicate fertilized eggs. Gonads are outlined in white. Scale bar: 10 µm.

Genetic mosaic worms lacking *vpr-1* in the somatic gonad are fertile (Fig. 4), consistent with *vpr-1* acting non-autonomously in these cells. If MSPd signaling promotes gonadogenesis, then overexpressing *vpr-1* in various tissues should rescue the *vpr-1* null defect. To test this hypothesis, we first drove a *vpr-1* genomic fragment containing introns and 3′ UTR (*vpr-1g*) in the germ line using the *pie-1* promoter (*pie-1p::vpr-1g*). Single-copy integrants on chromosome II were used to avoid germline silencing mechanisms. Two independently generated integrated transgenes completely rescued the *vpr-1(tm1411)* gonad and body wall muscle mitochondrial defects (Fig. 5A-D) (Schultz et al., 2017), and are likely to contribute maternal and zygotic *vpr-1*. To test whether zygotic *vpr-1* germline expression is sufficient for rescue, we crossed *vpr-1* null mutant males expressing *pie-1p::vpr-1g* to *M+ Z− vpr-1* hermaphrodites. The resulting *M−* zygotic germline *vpr-1^+^* mutant progeny were largely fertile with proliferating germ cells and differentiating gametes (Fig. 5B). The ∼20% of mutant hermaphrodites that were sterile exhibited a novel phenotype whereby oocytes differentiate near the vulva, a defect apparently due to delayed spermathecal and uterine development (Fig. 5E). As sheath cells are essential for germ cell proliferation, meiosis and ovulation (Hubbard and Greenstein, 2000; Killian and Hubbard, 2005; McCarter et al., 1997), sheath development must occur in the rescuing *vpr-1* mutant *pie-1p::vpr-1g* transgenics. These data suggest that germline *vpr-1* expression is sufficient to promote gonadogenesis in *vpr-1* null mutants. An important difference between this experiment and the mosaics is that the latter contained the entire *vpr-1* genomic region in an extrachromosomal transgene (see Discussion).

A caveat with integrated transgenes is that ectopic expression in unwanted tissues may result from surrounding regulatory sequences. To avoid this issue, we tested a panel of well-characterized neuron-specific promoters using high-copy extrachromosomal arrays (Kelly et al., 1997). Overexpressing *vpr-1g* throughout the nervous system using the *unc-119* promoter (Maduro and Pilgrim, 1995) largely rescues the *vpr-1(tm1411)* gonad defects (Fig. 5B,F). Similar results are observed using the *glr-5* promoter, which drives expression in ∼56 interneurons (Brockie et al., 2001), the *unc-25* promoter, which drives expression in 26 GABAergic motor neurons (Jin et al., 1999), and the *unc-17* promoter, which drives expression in ∼80 cholinergic neurons (Lickteig et al., 2001) (Fig. 5B,G). These overexpressing transgenic lines were fertile and could be maintained in a *vpr-1(tm1411)* background. A small percentage (<20%) of transgenic mutants exhibited the novel gonad phenotype described above. In addition to the neuronal promoters, overexpressing *vpr-1g* specifically in the intestine using the *ges-1* promoter (Kennedy et al., 1993) also rescues the *vpr-1* mutant gonad defect (Fig. 5B). The body wall muscle-specific *myo-3* promoter (Okkema et al., 1993) and hypodermis-specific *rol-6* promoter (Kramer et al., 1990) are sufficient for partial rescue (Fig. 5B), but the transgenic lines could not be stably propagated – in contrast to the mutant lines expressing *vpr-1* in neurons, the intestine or germ line. Body wall muscle and hypodermis may have limited capacity for *vpr-1* expression or MSPd secretion. *vpr-1* introns and 3′ UTR appear to contain key regulatory sequences, as transgenes containing the *vpr-1* cDNA and *unc-54* 3′ UTR exhibit less activity (data not shown). These results show that *vpr-1* overexpression in diverse cell types is sufficient to promote gonadogenesis.

The VPR-1 MSPd has been shown to interact with two broadly expressed receptors, CLR-1 and VAB-1, as well as an unidentified receptor(s) (Han et al., 2013, 2012; Tsuda et al., 2008). *clr-1* encodes a Lar-like receptor protein tyrosine phosphatase that is essential for larval fluid homeostasis and survival (Kokel et al., 1998). In adults, CLR-1 acts in body wall muscle to regulate actin remodeling and mitochondrial localization. The MSPd antagonizes muscle CLR-1 signaling to target mitochondrial tubules to myofibrils (Han et al., 2012). We considered the possibility that this pathway might constitute a checkpoint mechanism for gonadogenesis. Although *clr-1(e2530)* null and *clr-1(e1745ts)* temperature-sensitive mutants have degenerating gonads (Fig. S1), genetic mosaic and transgenic expression studies indicate that this degenerative defect is a consequence of hypodermal *clr-1* loss, which causes massive fluid accumulation and larval lethality (Fig. S1) (Kokel et al., 1998). Adult *vpr-1(tm1411) clr-1 RNAi* hermaphrodites and transgenic *vpr-1(tm1411); clr-1(e1745ts)* hermaphrodites expressing *clr-1* in the hypodermis exhibit correct muscle mitochondrial localization, yet have arrested gonads (Fig. S2A-D). Therefore, the *clr-1* muscle pathway is unlikely to influence gonadogenesis. The MSPd also antagonizes the VAB-1 Eph receptor, which is expressed throughout the nervous system (George et al., 1998; Miller et al., 2003; Tsuda et al., 2008). *vab-1(dx31); vpr-1(tm1411)* double-null mutants have arrested gonads (Fig. S2E), indicating that excess VAB-1 signaling does not cause arrested gonadogenesis. Collectively, the results are consistent with MSPd signaling being important for gonad development, but independent of excess CLR-1 or VAB-1 signaling individually.

### *vpr-1* and Notch act in parallel genetic pathways

**Fig. 6.**
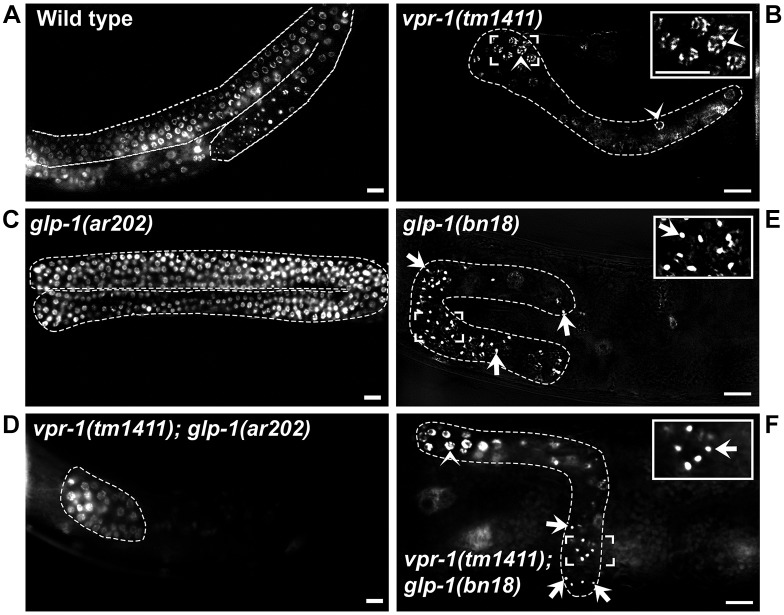
***vpr-1* and Notch act in parallel genetic pathways.** Deconvolved *z*-scans of (A) wild-type and (B-F) single- or double-mutant hermaphrodites of the indicated genotype stained with DAPI. Dashed white lines outline the gonads. *glp-1* encodes the Notch receptor. The *glp-1(ar202)* allele is a temperature-sensitive gain-of-function mutation, whereas the *glp-1(bn18)* allele is a temperature-sensitive loss-of-function mutation. All hermaphrodites were grown at 25°C. Insets are magnified views of the indicated regions. Arrowheads indicate germ cell nuclei and arrows indicate condensed sperm nuclei. Scale bars: 10 µm.

Signals from SS blast cells and the DTCs act in independent pathways to promote germline expansion during larval development (Hubbard and Greenstein, 2000; Killian and Hubbard, 2005; McCarter et al., 1997). Although the sheath pathway is not well understood, Notch signaling from the DTCs to germline stem cells controls proliferation and maintains stem cell fate (Hansen and Schedl, 2013; Kimble and Crittenden, 2005). Loss of the *glp-1* Notch receptor causes germ cells to exit mitosis and differentiate into sperm. By contrast, gain of *glp-1* function causes unregulated germ cell mitosis. To test whether *glp-1* acts in the same pathway as *vpr-1*, we first generated *vpr-1(tm1411); glp-1(ar202ts)* double mutants. *glp-1(ar202ts)* is a temperature-sensitive (ts) gain-of-function mutation (Pepper et al., 2003). DAPI and fluorescence deconvolution microscopy were used to visualize germ cell nuclei in wild-type, *vpr-1(tm1411)* single-mutant, *glp-1(ar202ts)* single-mutant, and *vpr-1(tm1411); glp-1(ar202ts)* double-mutant hermaphrodites grown at the restrictive temperature (25°C) from L1 (Fig. 6A-D). *vpr-1(tm1411); glp-1(ar202ts)* gonads (Fig. 6D) lack the germ cell tumor-like phenotype seen in *glp-1(ar202ts)* gonads (Fig. 6C) and instead closely resemble *vpr-1(tm1411)* gonads (Fig. 6B). This result indicates that *vpr-1* either acts downstream of *glp-1* or in an independent parallel pathway.

*glp-1(bn18ts)* is a temperature-sensitive loss-of-function mutation (Kodoyianni et al., 1992). Gonads from both *glp-1(bn18ts)* and *vpr-1(tm1411)* single mutants are small, but with an important difference. Germ cells in *glp-1(bn18ts)* gonads differentiate as sperm, whereas germ cells in *vpr-1(tm1411)* gonads fail to differentiate. If GLP-1 signaling is independent of *vpr-1*, then *vpr-1(tm1411); glp-1(bn18ts)* double-mutant gonads should contain sperm. To test this prediction, we grew single- and double-mutant hermaphrodites at 25°C from L1, stained them with DAPI, and performed fluorescence deconvolution microscopy. Characteristic highly condensed sperm chromosomes are not observed in *vpr-1(tm1411)* gonads (Fig. 6B), but are clearly observed in *glp-1(bn18ts)* single-mutant (Fig. 6E) and *vpr-1(tm1411); glp-1(bn18ts)* double-mutant (Fig. 6F) gonads. This result indicates that GLP-1 signaling prevents sperm differentiation in *vpr-1* mutants. These genetic data support the model that *vpr-1* and *glp-1* act in independent genetic pathways to promote germ cell expansion and differentiation.

### Neuronal *vpr-1* activity is crucial shortly after embryo hatching

**Fig. 7.**
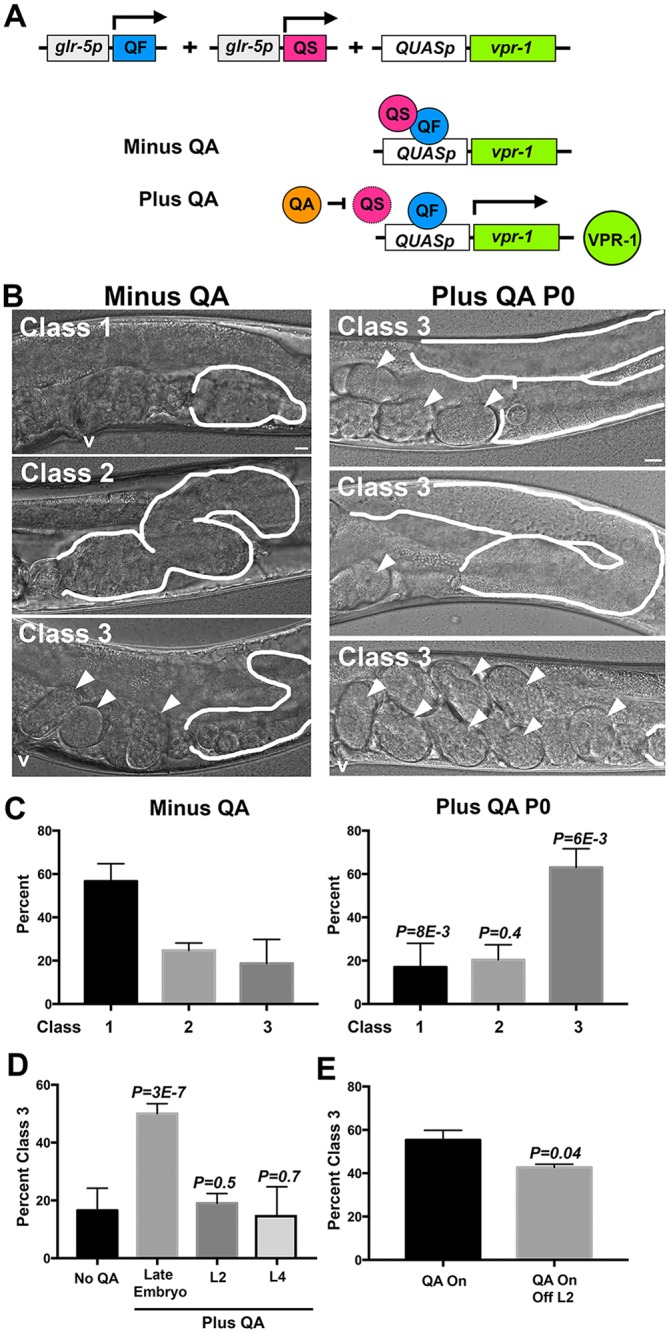
**Neuronal *vpr-1* expression is sufficient for gonadogenesis early in postembryonic development.** (A) The Q system was used to drive *vpr-1* expression in head interneurons under the *glr-5* promoter. Quinic acid (QA) is used to inhibit QS repressor activity and drive *vpr-1* expression. See main text for an explanation of the system. (B) DIC images of transgenic *vpr-1* mutants grown in the absence and presence of QA. When mutant adults (P0) lay eggs on QA plates, the F1 progeny are largely fertile. Class 1 gonads show lack of germ cell expansion and gamete development. Class 2 gonads show developing oocytes, but lack fertilized eggs. Class 3 gonads show gamete development and fertilized eggs. Gonads are outlined in white. Arrowheads indicate fertilized eggs. v, vulva. Scale bar: 10 µm. (C) Quantification of classes from A minus and plus QA. (D) Percentage class 3 mutant adults following QA addition at various times during development. The late embryo stage consisted of embryos and hatching L1 larva. L2 and L4 larval stages are also shown. (E) Percentage class 3 mutant adults comparing QA exposure throughout development (QA On) with exposure up to the L2 stage (Off L2). Data are from three or more independent experiments. Other transgenic lines produced similar results. The eggshell is a barrier to small molecules and QA exposure to worms is expected to occur at hatching. Error bars indicate s.d. Trial number is from 5-6 independent experiments. *P-*values were computed by two-tailed Student's *t*-test.

SS blast cells form during L1 and early L2, but do not complete development until mid to late L4 (Hubbard and Greenstein, 2000; Killian and Hubbard, 2005). To determine the critical time period for *vpr-1* activity we used the Q system, a drug-inducible binary gene expression system (Potter et al., 2010; Wei et al., 2012). The QF transcriptional activator binds a 16 bp sequence called *QUAS* to activate gene transcription (Fig. 7A). A transcriptional repressor called QS blocks *QUAS*-dependent transcription mediated by QF. The small molecule quinic acid (QA), which is added to plates, inhibits QS repressor activity, thereby activating gene expression. We used the *glr-5* promoter to drive QF and QS expression in ∼56 interneurons (Fig. 7A). Seven independent transgenic lines were generated expressing *glr-5p::QF*, *glr-5p::QS* and *QUASp::vpr-1g* in *vpr-1(tm1411)* mutants (see Materials and Methods). Six lines grew robustly in the presence of QA. All seven lines exhibited minimal QA-independent *vpr-1* expression, as indicated by gamete development in a small percentage of transgenic *vpr-1(tm1411)* hermaphrodites. The Q system was more tightly regulated than the heat shock promoter (data not shown). Providing QA produced functional VPR-1, as evidenced by rescue of the muscle mitochondrial defect (Fig. S3). We selected line 3 for further characterization because it grew very slowly without QA but grew rapidly with QA. An advantage of this line is that sufficient numbers of transgenic *vpr-1(tm1411)* homozygotes could be generated for staging. Similar results were observed for lines that failed to grow without QA.

Three phenotypic classes were used to quantify the data (Fig. 7B). Class 1 gonads lack germ cell expansion and gamete differentiation, closely resembling nontransgenic *vpr-1* mutant gonads. Class 2 gonads contain visible oocytes or an expanded germ cell population, but are sterile with no fertilized eggs in the uterus. Class 3 gonads contain differentiated gametes and fertilized eggs. In the absence of QA, 57% of transgenic *vpr-1* mutant gonads were class 1 and 19% were class 3 (Fig. 7C). When transgenic mutant adults (P0) were added to QA plates, their adult F1 progeny contained 17% class 1 gonads and 63% class 3 gonads (Fig. 7C). These class 3 gonads were larger and contained more fertilized eggs than class 3 gonads from worms lacking QA (Fig. 7B). The number of class 2 gonads did not significantly change in the presence or absence of QA. Therefore, inducing *vpr-1* expression in head interneurons throughout development is sufficient to promote gonadogenesis.

Next, we exposed worms at different stages of development to QA plates and analyzed their gonads 3 days later. Adding late-stage embryos or fresh hatchlings to QA plates is sufficient to induce a 3-fold increase in class 3 gonad percentage (Fig. 7D). However, adding L2 stage or older worms to QA plates did not affect gonadogenesis. These data suggest that *vpr-1* activity is needed shortly after embryogenesis, when QA first becomes available to hatchlings. Maternal *vpr-1* mRNA provided in *vpr-1(tm1411)* embryos is sufficient for gonad induction. As this maternal contribution is not renewable, we considered the possibility that *vpr-1* is sufficient transiently, early in development. To investigate this idea further, we provided QA to transgenic *vpr-1* mutant adults, let their progeny hatch on QA plates, and then moved the L2 worms to plates lacking QA. We found little difference in class 3 gonad percentage in these experiments compared with controls grown continuously on QA (Fig. 7E). These data support the model that *vpr-1* activity is crucial for gonadogenesis during a short time period between hatching and the early L2 stage.

## DISCUSSION

VAPs are broadly expressed proteins that function as secreted signaling molecules and intracellular scaffolds between the endoplasmic reticulum and other cytoplasmic structures (Dong et al., 2016; Han et al., 2013, 2012; Lev et al., 2008; Stoica et al., 2016; Tsuda et al., 2008). The first function often requires VAP synthesis in cells distinct from the effector cells (i.e. a non-cell-autonomous function), whereas the second, housekeeping function requires VAP synthesis only in the effector cell. The extent to which these diverse functions contribute to animal development and physiology is an open question. Here we present evidence in *C. elegans* that VPR-1 acts as a permissive signal for postembryonic gonadogenesis. Taken together with previous studies, our results support the model that neurons, germ cells, and intestinal cells secrete the MSPd into the pseudocoelom. A key signaling target appears to be somatic gonadal sheath cell precursors, which are essential for germ cell expansion and differentiation during larval development. Below we discuss evidence for this model, along with implications for reproduction and motor neuron diseases.

*vpr-1* mutants are maternal effect sterile due to arrested gonadogenesis. We found that germ cells fail to expand and differentiate into sperm or oocytes during larval development. Importantly, the somatic gonad is also affected, as the SS lineages that form the sheath cells and spermatheca do not fully proliferate and differentiate. Genetic mosaic and transgenic overexpression studies indicate that the *vpr-1* mutant SS lineage defect is non-cell-autonomous. Previous SS laser ablations showed that these cells are crucial for germ cell expansion and gametogenesis (Killian and Hubbard, 2005; McCarter et al., 1997). By contrast, laser or genetic ablation of germ cells does not appear to impact somatic gonad development (Beanan and Strome, 1992; Kimble, 1981; Sulston et al., 1983). The *vpr-1* mutant gonad defect could be largely explained by abnormal SS, or SS precursor, cells. Consistent with this idea, SS blast cells (McCarter et al., 1997) and *vpr-1* act in parallel to GLP-1 Notch receptor signaling to regulate germ cell proliferation and differentiation. There are differences between SS-ablated and *vpr-1* mutant gonads. In particular, germ cells enter meiotic pachytene following SS ablation and sometimes differentiate into sperm (McCarter et al., 1997), whereas germ cell nuclei in *vpr-1* mutants do not show pachytene or sperm chromosomal morphologies. Timing in these experiments could be important. Neuronal *vpr-1* expression is sufficient to induce gonadogenesis shortly after embryo hatching, but not after the L1 stage. The SS ablations were performed at the L2-L3 molt (Killian and Hubbard, 2005; McCarter et al., 1997). VPR-1 could also have an important role in the germ line that is independent of somatic gonadal cells. In any event, our results are consistent with the VPR-1 MSPd acting as a permissive signal at least in part on the developing somatic gonad.

Genetic mosaics indicate that zygotic *vpr-1* activity is important in the nervous system, germ line and intestine for fertility. Transgene loss in either the AB (primarily neuronal) or P4 (germ line) lineage caused gonads to arrest, although gonad development often appeared more advanced than that of gonads in *M− Z− vpr-1* animals. Intestinal *vpr-1* loss also caused sterility in some animals. Gonads from these mosaics produced oocytes but exhibited ovulation defects or other defects consistent with an abnormal somatic gonad. Our mosaics used fosmid DNA containing the entire *vpr-1* genomic locus and likely most gene regulatory sequences. Although transgenes are typically silenced in the germ line due to their repetitive nature (Kelly et al., 1997), fosmids are more likely to at least partially escape germline silencing and better reflect endogenous gene expression (Govindan et al., 2009; Yochem and Herman, 2003). However, our mosaic lines lacked sufficient maternal *vpr-1* to induce gonadogenesis and therefore provided a zygotic *vpr-1* activity readout. Using smaller DNA transgenes to overexpress *vpr-1* specifically in neuron subsets, the intestine or germ cells was sufficient to induce gonadogenesis. These transgenes lacked many endogenous gene regulatory sequences and contained promoter fragments that drive high-level expression. Including *vpr-1* introns and 3′ UTR in the constructs enhanced fertility in the transgenic lines, allowing for stable propagation in a *vpr-1* null background. Based on these data we speculate that, in wild-type hermaphrodites, multiple cell types contribute to a secreted MSPd pool. Neurons, germ cells and maternal *vpr-1* mRNA might be particularly important sources early in postembryonic development. As germ cell ablation does not impact somatic gonad development (Beanan and Strome, 1992; Kimble, 1981; Sulston et al., 1983), we consider it likely that there is some redundancy among MSPd secretion cell types. At present, genes that are essential for MSPd processing and secretion have yet to be discovered.

An important missing element to the VPR-1 secretion model is the MSPd receptor, which is presumably expressed on SS blast or precursor cells. The MSPd receptors VAB-1 and CLR-1 are both expressed in gonadal and peripheral tissues (Brisbin et al., 2009; George et al., 1998; Han et al., 2012; Kokel et al., 1998). However, these receptors individually do not appear to mediate the (early) role of VPR-1 in gonadogenesis, as *vab-1* loss or *clr-1* loss does not suppress the *vpr-1* null mutant gonadogenesis defect. It is possible that eliminating both receptors together could be suppressive, but the triple mutants are likely to exhibit severe embryonic lethality. We consider it unlikely that muscle relays a metabolic checkpoint signal to the gonad, because suppressing the *vpr-1* mutant muscle mitochondrial defect does not trigger gonadogenesis. During adulthood, MSPs secreted by sperm induce oocyte maturation via gonadal sheath cell heteromeric G-protein pathways (Ellis and Stanfield, 2014; Govindan et al., 2006). Extracellular VPR-1 MSPd binds to sheath cells and is sufficient to induce oocyte maturation in the absence of sperm (Tsuda et al., 2008). Therefore, a similar signaling mechanism might also mediate VPR-1 MSPd signaling earlier in development. Additional work is necessary to clarify the mechanism by which VPR-1 induces gonadogenesis.

Why might animal VAPs have such disparate biochemical functions? Animals require signaling mechanisms to coordinate tissue development and physiology. These mechanisms are well conserved in diverse animals, but typically not in unicellular eukaryotes, multicellular fungi or plants (Richter and King, 2013; Srivastava et al., 2010). Ligands and receptors appear to have emerged early in animal evolution through gene duplication, followed by rearrangements and other mutations (Braasch et al., 2009; Markov et al., 2008). In this scenario, the duplication event frees up one gene copy to acquire a new signaling function. The other gene copy continues to perform its original function. However, there might be circumstances when a gene copy retains both functions. We speculate that VAPs are an example of the latter. As the VAP signaling function depends on MSPd cleavage and secretion, it seems likely that the housekeeping function is destroyed. Cells could help overcome this problem by increasing VAP expression, redundancy, or tightly regulating secretion.

The MSPd has functional similarities to testosterone, albeit without the sex-specific features. Both molecules are inducers of gonadogenesis and have metabolic effects on striated muscle (Han et al., 2013, 2012; Kadi, 2008; Wang et al., 2009). The biggest similarity lies in their role in lower motor neuron (LMN) degeneration. ALS8 patients contain a P56S substitution in the VAPB MSPd and present with ALS, atypical ALS, or late-onset SMA (Marques et al., 2006; Nishimura et al., 2004). CAG trinucleotide repeat expansion in the androgen receptor causes spinal bulbar muscular atrophy (SBMA) (La Spada et al., 1991). A common theme in ALS8 and SBMA disorders is the degeneration of LMNs in the spinal cord anterior horn. Recent mouse studies provide compelling evidence that skeletal muscle is crucial to SBMA pathogenesis (Cortes et al., 2014; Katsuno et al., 2012; Lieberman et al., 2014; Monks et al., 2007; Musa et al., 2011). A Cre recombinase strategy to excise polyglutamine-expanded androgen receptor specifically from muscle prevents weight loss, aberrant motor phenotypes, muscle pathology and motor neuronopathy, as well as dramatically extends survival (Cortes et al., 2014). Testosterone, which is primarily produced in the Leydig cells of the testis, is crucial for SBMA pathogenesis. ALS8 and SBMA patients could have a significant skeletal muscle pathogenic component that originates in part from gonadal tissues. Further work on VAPB and its paralog VAPA could shed new light on the relationship between the reproductive system and motor neuron survival.

## MATERIALS AND METHODS

### *C. elegans* genetics and strains

*C. elegans* were maintained at 20°C unless otherwise indicated, and fed with NA22 *E. coli* (Brenner, 1974; Edmonds et al., 2010; Kubagawa et al., 2006). The following strains were used: N2 Bristol (wild type); VC1478 *vpr-1(tm1411)/hT2 [bli-4(e937) let-?(q782) qIs48] (I; III)*; JK2868 *qIs56 [lag-2p::GFP* *+ unc-119(+)]*; WS2170 *opIs110 [lim-7p::YFP::act-5+unc-119(+)]*; OD58 *unc-119(ed3) III; ltIs38 [pie-1p::GFP::PH(PLC1delta1)+unc-119(+)]*; DG1575 *tnIs6 [lim-7p::GFP+rol-6(su1006)]*; CB4108 *fog-2(q71)*; CZ337 *vab-1(dx31)*; GC833 *glp-1(ar202)*; DG2389 *glp-1(bn18)*; XM1101 *vpr-1(tm1411)/hT2 [bli-4(e937) let-?(q782) qIs48]; clr-1(e1745ts)*; XM1102 *clr-1(e2530)/mIn1 [dpy-10(e128) mIs14]*; XM1103 *vpr-1(tm1411)/hT2 [bli-4(e937) let-?(q782) qIs48]; vab-1(dx31)*; XM1104 *vpr-1(tm1411)/hT2 [bli-4(e937) let-?(q782) qIs48]; glp-1(ar202)*; Xm1105 *vpr-1(tm1411)/hT2 [bli-4(e937) let-?(q782) qIs48]; glp-1(bn18)*.

Studies with the *clr-1(e1745)*, *glp-1(ar202)* and *glp-1(bn18)* temperature-sensitive alleles were conducted at permissive (16°C) and restrictive (25°C) temperatures, as indicated. Strain construction was performed using PCR, sequencing and phenotypic analyses. *vpr-1(tm1411)* mutants are maternal effect sterile. Phenotypes were evaluated in *vpr-1(tm1411)* homozygous F2 progeny from *vpr-1(tm1411)/hT2* heterozygotes (P0), unless otherwise indicated. *vpr-1(tm1411)* homozygous F1 progeny contain maternal *vpr-1* mRNA. To investigate zygotic *vpr-1* activity, progeny of fertile F1 *vpr-1* mutants mated to wild-type males were examined. RNAi was performed by the feeding method (Timmons and Fire, 1998). HT115(DE3) bacterial feeding strains were obtained from the genome-wide library (Kamath et al., 2003). PCR and sequencing (UAB Heflin Center for Genomics Sciences) were used to confirm that strains contained the correct clones.

### Molecular cloning

Pan-neuronal (*unc-119p*, 2000 bp) (Maduro and Pilgrim, 1995), GABA motor neuron (*unc-25p*, 1893 bp) (Jin et al., 1999), cholinergic motor neuron (*unc-17p*, 2003 bp) (Lickteig et al., 2001), head interneuron (*glr-5p*, 20003 bp) (Brockie et al., 2001), intestine (*ges-1p*, 2003 bp) (Kennedy et al., 1993), body wall muscle (*myo-3p*, 2385 bp) (Okkema et al., 1993) and hypodermal (*rol-6p*, 2000 bp) (Kramer et al., 1990) promoters were fused upstream of the *vpr-1* genomic locus, which included 659 bp of the 3′ UTR (collectively termed *vpr-1g*). This *vpr-1* genomic fragment enhanced rescue of the *vpr-1* mutant gonadogenesis defect relative to the *vpr-1* cDNA fused to the *unc-54* 3′ UTR. PCR was used to amplify sequences from genomic DNA. The *unc-119p::vpr-1g*, *unc-25p::vpr-1g*, *unc-17p::vpr-1g*, *glr-5p::vpr-1g* and *ges-1p::vpr-1g* constructs were generated using PCR and restriction enzymes in a TOPO vector backbone. The *rol-6p::vpr-1g* and *myo-3p::vpr-1g* constructs were generated using Gibson assembly (New England Biolabs) in a pGEM backbone. Primers are shown below. The *pie-1p::vpr-1g* construct was generated by Knudra Transgenics. The *pie-1* promoter sequence included 1095 bp upstream of the translational start site. The *vpr-1* DNA sequence included exons and introns, as well as 745 bp of the 3′ UTR.

To create the Cas9 DNA template for tdTomato insertion into the *clr-1* genomic locus, Gibson assembly was used to construct a plasmid containing *clr-1* 2 kb left homology arm::tdTomato::*clr-1* 3′ UTR::*C. briggsae unc-119*::2 kb right homology arm. The single guide RNA (sgRNA) plasmid was derived from Addgene plasmid 46169. Cas9 targeting sequence was 5′-ACTATATCTCTAAGACATAT-3′. PCR was used to amplify the entire sgRNA backbone, except for 20 bp from *unc-119*. DNA fusions were constructed using Gibson assembly. The *rol-6p::clr-1* construct was made with *clr-1* genomic DNA. 2 kb upstream of the *rol-6* start codon was amplified by PCR. All constructs were confirmed by sequencing (UAB Heflin Center for Genomics Sciences). Primers (5′-3′; F, forward; R, reverse) were: vpr-1 F1 sacII, GGGGACAACTTTCCGCGGAAAAAAATGTCTGAAAAGCACAGTCTTCTG; vpr-1 R1 kpnI, GGGGACTGCTTTGGTACCCCGAGATAATACGGCGAAAA; glr-5 F1 BHI NtI, GGGGACAACTTTGGATCCGCGGCCGCGTCACAATTTTCGGGTGTCGTAG; glr-5 R1 NeI ScII, GGGGACTGCTTTCCGCGGGCTAGCGATGCTTATTATTCACATGTTTCAAACC; unc-17 F1 BHI NEI, GGGGACAACTTTGGATCCAGCGGCCGCTTCACACAATTAAGAATTTTAAGATTTGGG; unc-17 R1 NeI ScII, GGGGACTGCTTTCCGCGGGCTAGCCTCTCTCTCTCCCCCTGGAATATT; ges-1 F1 BHI NtI, GGGGACAACTTTGGATCCAGCGGCCGCAAACTCCGAACTATGATGACGAA; ges-1 R1 NeI ScII, GGGGACTGCTTTCCGCGGGCTAGCCTGAATTCAAAGATAAGATATGTAATAGATTTTT; unc-25 F1 BHI NtI, GGGGACAACTGGATCCAGCGGCCGCGAGAAATAAGAAATAATTGTATAATTTTTTTTTC; unc-25 R1 ScII NeI, GGGGACTGCTTTCCGCGGGCTAGCTTTTGGCGGTGAACTGAGCTTTT; topo F1 KpnI, GGGGACAACTTTGGTACCCCTGAATGGCGAATGGAC; topo pR1 BHI, GGGGACTGCTTTGGATCCAGCTCACTCAAAGGCGGTAA; vpr-1 F1 NheI, GGGGACAACTTTGCTAGCAAAAAAATGTCTGAAAAGCACAGTCTTCTG; glr-5 R1 ScII NeI, GGGGACTGCTTTGCTAGCCCGCGGGATGCTTATTATTCACATGTTTCAAACC; F1 rol-6 pgem5, AGGTCGACCATATGGGAGAGCTAGAAAAACGATGGATTGAGTTATCTGG; R1 vpr-1 rol-6, AGACTGTGCTTTTCAGACATCTGGAAATTTTCAGTTAGATCTAAAGATATATCC; F2 rol-6 vpr-1, GATCTAACTGAAAATTTCCAGATGTCTGAAAAGCACAGTCTTCTG; R2 pgem vpr-1, CTATGCATCCAACGCGTTGGGAACCATAAACATCAAATTTTATTGTACCATATAC; F1 pmyo-3 pgem5, GGTCGACCATATGGGAGAGCTGGCTATAATAAGTTCTTGAATAAAATAATTTTCCC; R1 vpr-1 pmyo-3, GCAGAAGACTGTGCTTTTCAGACATTTCTAGATGGATCTAGTGGTCGTGG; F2 pmyo-3 vpr-1, CCACGACCACTAGATCCATCTAGAAATGTCTGAAAAGCACAGTCTTCTGC; R2 pmyo-3 pgem5, GCTATGCATCCAACGCGTTGGGAACCATAAACATCAAATTTTATTGTACCATATAC.

### Imaging

Microscopy images were taken by a motorized Zeiss Axioskop equipped with epifluorescence and AxioVision software version 4.8. For DAPI staining, worms were fixed in 10% neutral buffered formalin (Sigma-Aldrich), mixed with 0.5 µg/ml DAPI, and incubated for 24 h at 4°C. Worms were then washed five to eight times with sterile water and mounted for microscopy. To image gonads, axial scans were performed and out-of-focus light was removed with deconvolution software (AxioVision).

### Transgenics

To generate transgenic *C. elegans*, plasmids (5-60 ng/μl) were injected into wild-type or *vpr-1(tm1411)/hT2* young adult hermaphrodite gonads. The *myo-3p::mitoGFP* or *sur-5p::NLS-GFP* constructs were used for selection (Labrousse et al., 1999). Multiple independent transgenic lines were analyzed. The *pie-1p::vpr-1g* transgenic lines were generated using MosSCI single-copy insertion (*ttTi5605* Mos1 allele, near the center of chromosome II) by Knudra Transgenics. Integrated transgenes were crossed into the *vpr-1(tm1411)* background and maintained as transgenic *vpr-1* mutant homozygotes.

### Genetic mosaic analysis

Mosaic worms were generated by microinjecting 10 ng/µl WRM06B28 fosmid DNA containing the *vpr-1* genomic locus together with 10 ng/µl pTG96 (*sur-5p::NLS-GFP*) plasmid into *vpr-1(tm1411)*/*hT2* hermaphrodite gonads (Yochem et al., 1998). GFP expression was used to select transgenic lines. The *vpr-1*^+^ fosmid rescued the sterility, body wall muscle mitochondria defect, fat accumulation defect, and the slow growth of *vpr-1(tm1411)* null hermaphrodites (Han et al., 2013). To identify mosaics, ∼12,000 transgenic *vpr-1(tm1411)* worms were screened from three independent lines. *vpr-1^+^* loss in the AB, P1, P2, P3, P4, E, EMS and other lineages was scored as previously described (Han et al., 2013). Gonads in the transgenic worms were analyzed using DIC microscopy. The *vpr-1* mutant gonad defect is rescued by maternally deposited *vpr-1* mRNA. However, the mosaic strategy was successful because the rescuing fosmid lines did not supply sufficient *vpr-1* maternal mRNA, which was likely to be due to partial transgene silencing in the germ line. *vpr-1* mutant progeny lacking the transgene in all cells were sterile.

### CRISPR/Cas9

CRISPR/Cas9 methods were performed as previously published (Friedland et al., 2013). DNA template, sgRNA, Cas9 and *myo3p::mitoGFP* plasmids were injected into *unc-119(ed3)* worms. Progeny were screened for rescue of the *unc-119* movement defect and loss of *myo3p::mitoGFP*. Individual worms were isolated repeatedly to ensure 100% segregation. PCR and sequencing were used to confirm tdTomato insertion. The *clr-1::tdTomato* Cas9 line did not exhibit the fluid accumulation phenotype caused by reduced *clr-1* function (Kokel et al., 1998), indicating that tdTomato fusion does not affect CLR-1 activity.

### Binary Q-inducible gene expression

Q system plasmids *XW08 unc-4p::QF-SL2::mCherry::3′UTR-unc-54*, *XW09 unc-4p::QS-SL2::mCherry::3′UTR-unc-54* and *XW12 quasp::Δpes10::GFP::3′UTR-unc-54* were generously provided by Dr Xing Wei and Dr Kang Shen (Wei et al., 2012). Gibson assembly and Phusion high-fidelity DNA polymerase (New England Biolabs) were used to generate *glr-5p::QF-SL2::mCherry::3′UTR-unc-54*, *glr-5p::QS-SL2::mCherry::3′UTR-unc-54* and *quasp::Δpes10::vpr-1-SL2::GFP::3′UTR-unc-54* plasmids. The *vpr-1* sequence comprised 1739 bp, including 7 bp upstream of the translational start site, exons and introns, and 659 bp in the 3′ UTR. All constructs were confirmed by sequencing (UAB Heflin Center for Genomics Sciences). Primers were (5′-3′; F, forward; R, reverse): XW08 F1, CGGTTTGAAACATGTGAATAATAAGCATCATGGGCGCGCCTCTAGAGGATC; XW08 R1, GTTCTACGACACCCGAAAATTGTGACGCATGCAAGCTTGGCGTAATC; XW08 *glr-5* F1, GATTACGCCAAGCTTGCATGCGTCACAATTTTCGGGTGTCGTAGAAC; XW08 *glr-5* R1, GATCCTCTAGAGGCGCGCCCATGATGCTTATTATTCACATGTTTCAAACCG; XW09 F1, GGTTTGAAACATGTGAATAATAAGCATCATGGGCGCGCCTCTAGAGGATCC; XW09 R1, CTACGACACCCGAAAATTGTGACGGCCGGCCCAGTCAGTGCG; XW09 *glr-5* F1, CGCACTGACTGGGCCGGCCGTCACAATTTTCGGGTGTCGTAG; XW09 *glr-5* R1, GGATCCTCTAGAGGCGCGCCCATGATGCTTATTATTCACATGTTTCAAACC; XW12 F1, GGGAAACTGCTTCAACGCATCATGAGTAAAGGAGAAGAACTTTTCACTG; XW12 R1, GCAGAAGACTGTGCTTTTCAGACATTTTTTCTACCGGTACCGTCGAC; VPR-1 F1, GTCGACGGTACCGGTAGAAAAAATGTCTGAAAAGCACAGTCTTCTGC; VPR-1 R1, AGGTGAAAGTAGGATGAGACAGCAACCATAAACATCAAATTTTATTGTACCATATACA; SL2 F1, TGTATATGGTACAATAAAATTTGATGTTTATGGTTGCTGTCTCATCCTACTTTCACCT; SL2 R1,CAGTGAAAAGTTCTTCTCCTTTACTCATGATGCGTTGAAGCAGTTTCCC.

To generate transgenic *C. elegans*, *glr-5p::QF-SL2::mCherry::3′UTR-unc-54* (10 ng/μl), *glr-5p::QS-SL2::mCherry::3′UTR-unc-54* (50 ng/μl), *QUASp::Δpes10::vpr-1-SL2::GFP::3′UTR-unc-54* (10 ng/μl) and *myo-3p ::mito::GFP* (30 ng/μl) plasmids were injected into *vpr-1(tm1411)/hT2* young adult hermaphrodite gonads. Transgenic lines were selected based on *mCherry* and *myo3p::mito::GFP* expression. Seven independent lines were created. Six responded well to quinic acid (QA) treatment. For treatment, 300 µl 300 mg/ml pH 6.5 QA (Sigma-Aldrich) was mixed with 40 µl M9 buffer and added to NGM plates seeded with NA22 bacteria. All lines exhibited very low *vpr-1* expression in the absence of QA, partially rescuing the *vpr-1* mutant gonadogenesis defect in a small percentage of transgenic worms. Increasing the *glr-5p::QS* dosage in the transgenic arrays appeared to limit QA-independent *vpr-1* expression.

### Statistics

Two-tailed Student's *t*-tests were computed using Excel 2013 (Microsoft) without the assumption of equal variance.
